# Structural and Thermal Characterization of Bluepha^®^ Biopolyesters: Insights into Molecular Architecture and Potential Applications

**DOI:** 10.3390/ma17235863

**Published:** 2024-11-29

**Authors:** Magdalena Martinka Maksymiak, Silke Andrä-Żmuda, Wanda Sikorska, Henryk Janeczek, Paweł Chaber, Marta Musioł, Marcin Godzierz, Marek Kowalczuk, Grazyna Adamus

**Affiliations:** Centre of Polymer and Carbon Materials, Polish Academy of Sciences, 34. M. Curie-Skłodowska St., 41-819 Zabrze, Poland; sandra@cmpw-pan.pl (S.A.-Ż.); wsikorska@cmpw-pan.pl (W.S.); hjaneczek@cmpw-pan.pl (H.J.); pchaber@cmpw-pan.pl (P.C.); mmusiol@cmpw-pan.pl (M.M.); mgodzierz@cmpw-pan.pl (M.G.); mkowalczuk@cmpw-pan.pl (M.K.)

**Keywords:** Bluepha^®^ biopolyesters, poly((*R*)-3-hydroxyalkanoates), PHBH, structural characterization, ESI-MS/MS, thermal properties

## Abstract

This study presents an in-depth molecular and structural characterization of novel biopolyesters developed under the trademark Bluepha^®^. The primary aim was to elucidate the relationship between chemical structure, chain architecture, and material properties of these biopolyesters to define their potential applications across various sectors. Proton nuclear magnetic resonance (^1^H NMR) analysis identified the biopolyesters as poly[(*R*)-3-hydroxybutyrate-*co*-(*R*)-3-hydroxyhexanoate] (PHBH) copolymers, containing 4% and 10% molar content of hydroxyhexanoate (HH) units, respectively. Mass spectrometry analysis of PHBH oligomers, produced via controlled thermal degradation, further confirmed the chemical structure and molecular architecture of the PHBH samples. Additionally, multistage electrospray ionization mass spectrometry (ESI-MS/MS) provided insights into the chemical homogeneity and arrangement of comonomer units within the copolyester chains, revealing a random distribution of hydroxyhexanoate (HH) and hydroxybutyrate (HB) units along the PHBH chains. X-ray diffraction (XRD) patterns demonstrated partial crystallinity in the PHBH samples. The thermal properties, including glass transition temperature (*T_g_*), melting temperature (*T_m_*), and melting enthalpy (Δ*H_m_*), were found to be lower in PHBH than in poly(*R*)-3-hydroxybutyrate (PHB), suggesting a broader application potential for the tested PHBH biopolyesters.

## 1. Introduction

Poly(hydroxyalkanoates) (PHAs), synthesized through biotechnological methods, are high-molecular-mass biopolyesters that many microorganisms store intracellularly as carbon and energy reserves [1,2,3,4]. Due to their biodegradability and favorable thermomechanical properties, PHAs represent an interesting group of polymers [3,5]. The first of these advantages largely stems from the fact that bacteria capable of degrading PHA biopolyesters inhabit nearly all types of terrestrial and aquatic environments [6].

Moreover, widely studied biopolyesters within the PHA family, such as poly(3-hydroxybutyrate) (PHB) and the copolymer poly(3-hydroxybutyrate-*co*-3-hydroxyvalerate) (PHBV), have physical characteristics—including melting temperature, tensile strength, and Young’s modulus—comparable to those of polypropylene (PP) and low-density polyethylene (LDPE) [7,8]. Of significant importance is that PHA biopolyesters are thermoplastic, making them easily processable using methods typically applied to conventional thermoplastic polymers [9,10]. The simplest biopolyesters from the PHA family are characterized by high hydrophobicity (significant moisture resistance) and low permeability to oxygen and carbon dioxide, which is crucial for polymers intended for food packaging [11,12]. High biodegradability in environments where waste is commonly stored, mechanical properties similar to polypropylene or low-density polyethylene, and the possibility of reducing production costs through the use of renewable raw materials in PHA synthesis, all make PHAs a promising alternative to petrochemical-derived polymers [4,13,14]. It is worth noting that, in addition to their biodegradability, PHAs also exhibit remarkable biocompatibility with living cells and tissues, which determines the use of these polymers in the biomedical field [15,16,17,18]. Oligomeric poly(3-hydroxybutyrate) and its in vivo degradation product, 3-hydroxybutyric acid, are naturally present in various mammalian tissues, including human tissues [19,20]. Poly(3-hydroxybutyrate) forms ion channels in the cell membrane of eukaryotic cells, while 3-hydroxybutyric acid belongs to the ketone bodies [21]. Moreover, the degradation product of PHAs, 3-hydroxybutyric acid, is less acidic compared to lactic acid, the degradation product of polylactide (PLA), which lowers the risk of chronic inflammation and necrosis [22]. Although the implantation of PHAs induces an immune response as a foreign body, this often subsides quickly, leading to the host organism’s acceptance of the implant. As a result, PHAs often provoke a milder immune response than implants made from PLA, poly(lactide-*co*-glycolide) (PLGA), or polycaprolactone (PCL) [23,24].

Although the discovery of polyhydroxyalkanoates (PHAs) dates back to 1925 when Maurice Lemoigne identified their simplest form, poly(3-hydroxybutyrate) (PHB), it has since been shown that over 160 different hydroxyalkanoic acids are components of PHAs [25,26,27].

PHAs synthesized by bacteria are usually random copolyesters with varying comonomer compositions, which affect their physical properties. Depending on the structure and content of the constitutional units, PHA properties can range from rigid and brittle polymers to flexible materials. The chemical structure of the constitutional units, their mutual proportions, and the distribution of their sequences influence the mechanical, physical, and processing properties, as well as the biodegradation rate of PHAs. The chemical structure of these polyesters, and thus their physicochemical properties, can be controlled during biosynthesis by selecting appropriate microorganisms, choosing the carbon source as the microbial feedstock, and optimizing biosynthesis conditions [28]. These three parameters offer relatively broad possibilities for controlling the properties of the resulting biopolyesters. Nevertheless, various chemical methods have also been used to alter the chemical structure of PHAs and, thus, their physicochemical properties [29,30,31].

Despite the broad spectrum of potential materials, only a few PHA biopolyesters are currently produced on an industrial scale. Nevertheless, ongoing research aims to modify biotechnological processes to obtain PHAs with diverse physicochemical properties and to establish relationships between their structure, molecular architecture, and properties [32,33,34].

This paper presents the molecular and structural characterization of two samples of PHAs biopolyesters, produced under the trademark Bluepha^®^ and kindly provided by Binhai Coastal Industrial Park (Yancheng, China). The molecular and structural properties of these samples were analyzed using GPC (gel permeation chromatography), ^1^H NMR and XRD techniques. To explore potential future applications of these biopolyesters, detailed information about their molecular structure and the relationship between their architecture and properties was established. Multistage electrospray ionization mass spectrometry (ESI-MS/MS) was employed to assess chemical homogeneity and determine the chemical structure of the end groups. To accurately verify the molecular structure of the PHBHs under study via mass spectrometry, the molar masses of these biopolyesters were adjusted to align with the spectrometer’s measurement capabilities. The controlled thermal degradation method described in the manuscript facilitated the production of PHBHs with reduced molar masses (PHBH oligomers) while maintaining their chemical structure, composition, and the sequential distribution of comonomer units. This approach enabled the creation of ideal models, which were subsequently used for the structural analysis of PHBH. Additionally, the thermal properties of the poly[(*R*)-3-hydroxybutyrate-*co*-(*R*)-3-hydroxyhexanoate] samples were evaluated using DSC (differential scanning calorimetry) and TGA (thermogravimetric analysis) techniques.

## 2. Materials and Methods

### 2.1. Materials and Reagents

Two samples of PHAs biopolyesters produced under the trademark Bluepha^®^ were kindly provided by Binhai Coastal Industrial Park (Yancheng, China). The received samples were marked as PHBH4 and PHBH10, respectively. Samples PHBH4 and PHBH10 were provided in powder form and were used in studies without further purification.

Chloroform (CHCl_3_) (Sigma Aldrich, Saint Louis, MS, USA), Dowex 50WX8-100 hydrogen form (Sigma Aldrich, Steinheim, Germany), hexane (VWR Chemicals, Fontenay sous Bois, France), methanol (Sigma Aldrich, Merck, Darmstadt, Germany) and potassium bicarbonate (KHCO_3_) (VWR Chemicals, Solon, OH, USA) were used without further purification.

### 2.2. Oligomers Preparation via Thermal Degradation

PHBH oligomers were prepared via thermal degradation of high molar mass PHBH4 and PHBH10 copolymers induced by KHCO_3_ and marked as PHBH4T and PHBH10T [35]. The molar ratio of PHBH/KHCO_3_ was equal to 40. First, PHBH and KHCO_3_ were added into a round bottom flask with 10 mL methanol. Next, the mixture was stirred for 15 min and then the solvent was slowly evaporated at low temperature (around 30 °C) under vacuum. Further, thermal degradation was carried out in an oven at 180 °C for 25 min. After this time, the round bottom flask was left outside the oven to cool down. The resulting product was dissolved in chloroform and stirred until a homogenous solution was obtained. Next, Dowex 50WX8-100 hydrogen form, strongly acidic, 50–100 mesh was added to the mixture and stirred for 2 h. Finally, the Dowex 50WX8-100 was removed by filtration. The resulting solvent was precipitated in hexane. The obtained oligomers were analyzed by ^1^H NMR, GPC, ESI-MS/MS, XRD and DSC techniques.

### 2.3. Methods

#### 2.3.1. Nuclear Magnetic Resonance (^1^H NMR)

The ^1^H NMR spectra were recorded on a Bruker-Avance II Ultrashield Plus Magnets 600 MHz (Bruker BioSpin GmbH, Rheinstetten, Germany). The samples were dissolved in CDCl_3_. Tetramethylsilane (TMS) was used as an internal standard.

#### 2.3.2. Gel Permeation Chromatography (GPC)

The analyzed samples were dissolved in CHCl_3_ in order to prepare solutions with a concentration of 0.3% *w*/*v*. Next, the received solutions were transferred through a VISCOTEK VE 1122 solvent delivery system (Malvern Panalytical Ltd., Malvern, UK) at 35 °C with a flow rate of 1 mL/min. For the analysis, two Mixed C Styragel columns (Agilent Technologies, Inc., Santa Clara, CA, USA) including a mixed bed (M_w_ = 200–2,000,000) and refractive index detector (Shodex SE 61, Showa Denko, Munich, Germany) were used. To create a calibration curve (to determine the molecular mass and molecular mass distribution of the analyzed PHAs samples), narrow molar-mass dispersity polystyrene standards from EasiCal^®^ Pre-prepared Calibration Kits (obtained from Agilent Technologies, Inc., Santa Clara, CA, USA) were applied.

#### 2.3.3. Differential Scanning Calorimetry (DSC)

The thermal properties of plain PHBH biopolyester samples and their corresponding oligomer samples were analyzed using a non-modulated differential scanning calorimeter (TA-DSC Q2000, TA Instruments, Newcastle, DE, USA) with a nitrogen flow rate of 50 mL min^−1^. To generate the DSC curves, the samples were heated from −50 °C to 200 °C at a rate of 20 °C min^−1^.

#### 2.3.4. Elemental Analysis

The elemental analysis was performed with an elemental analyzer VARIO EL III ELEMENTAR (Hanau, Germany) in an oxygen atmosphere (99.995%) with automatically optimized sample burning time. The thermal conductivity detector and multi-point calibration were used. The weighments were from 1 to 200 mg, with determination accuracy < 0.1%.

#### 2.3.5. X-Ray Diffraction (XRD)

The X-ray diffraction patterns were measured on a D8 advanced diffractometer (Bruker AXS, Karlsruhe, Germany) with a Cu–Kα cathode (λ = 1.54 Å). The scan rate was set to 0.5°/min and the scan step was 0.02° in the range of 5-80° 2Θ. To identify the fitting phases, the DIFFRAC.EVA program supported with the ICDD PDF#2 database was used. The peak decomposition method was applied to calculate the crystallinity of the analyzed samples.

#### 2.3.6. Thermogravimetric Analysis (TGA)

For the thermogravimetric analysis (TGA), a TGA/DSC1 Mettler-Toledo (Columbus, OH, USA) thermal analyzer was used. The studies were performed in the temperature range from room temperature to 800 °C with a heating rate of 10 °C/min in a stream of N_2_ (60 mL/min). To analyze the data obtained during the thermogravimetric analysis, the Mettler-Toledo Star System SW 9.30 was used.

#### 2.3.7. Electrospray Mass Spectrometry (ESI-MS/MS) Analyses

Electrospray ionization mass spectra were obtained by directly infusing the polymer samples into the ESI source of a Thermo LCQ Fleet ion-trap mass spectrometer (Thermo Fisher Scientific Inc., San Jose, CA, USA) using a syringe pump at a flow rate of 10 mL min^−1^. Nitrogen served as the nebulizing gas. The ESI source was maintained at 4.8 kV, with the capillary temperature set to 200 °C. The polymer samples were diluted in a 1:1 (*v*/*v*) chloroform/methanol mixture. For fragmentation studies, the ions of interest were isolated as monoisotopic peaks within the ion trap and activated through collision. Helium, present in the mass analyzer, functioned as the collision gas and also as an auxiliary gas. In ESI MS/MS analyses, precursor ions were selected in the ion trap and then activated through collision-induced dissociation. All measurements were carried out in the positive ion mode.

## 3. Results and Discussion

**Scheme 1:**
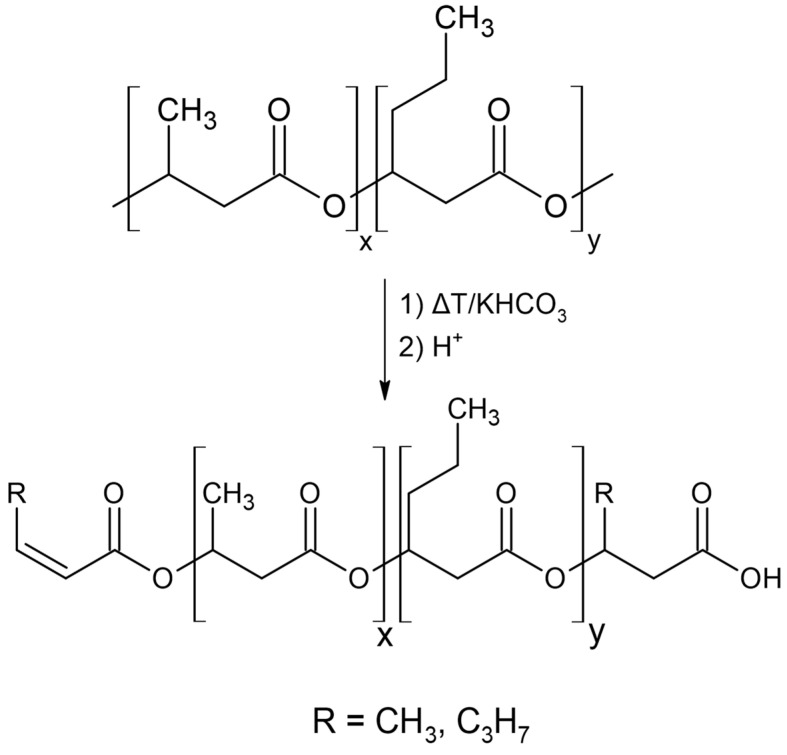
Thermal degradation of poly(3-hydroxybutyrate-*co*-3-hydroxyhexanoate) biopolyester.

The objects of the studies were two samples of poly[(*R*)-3-hydroxybutyrate-*co*-(*R*)-3-hydroxyhexanoate] biopolyesters (Bluepha^®^) marked as PHBH4 and PHBH10. Both samples were provided in powder form. Elemental analysis showed that the nitrogen content of both samples was lower than 0.06%, which confirmed that both PHBH biopolyester samples were free from biological residues. For the purpose of structural characterization of the biopolyesters studied, their oligomers were obtained by controlled carboxylate-induced thermal degradation (Scheme 1) [35,36].

The high molar mass PHBH4 and PHBH10 biopolyester samples and their corresponding oligomers were analyzed using gel permeation chromatography (GPC). The GPC results are detailed in Table 1.

Thermal properties of PHBH4 and PHBH10 biopolyesters and their respective oligomers obtained via thermal degradation were determined by DSC and TGA techniques. The DSC technique was used for determination of glass transition temperature *T_g_*, melting temperature *T_m_*, and melting enthalpy Δ*H_m_* [37]. The DSC analysis results are summarized in Table 2. Decomposition curves and maximum decomposition temperature rate *T_max_* of the biopolyester samples were determined with the aid of the TGA technique.

**Figure 1:**
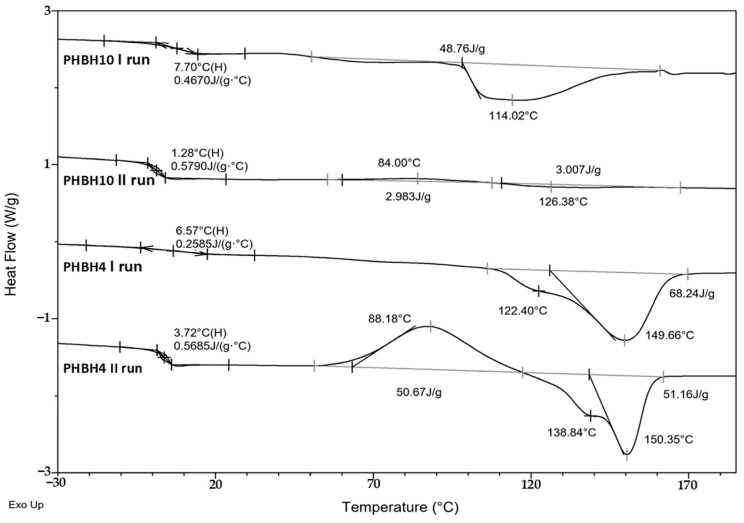
DSC traces (first and second runs) of the PHBH4 and PHBH10 biopolyesters.

The DSC traces of the PHBH biopolyesters are shown in Figure 1.

**Figure 2:**
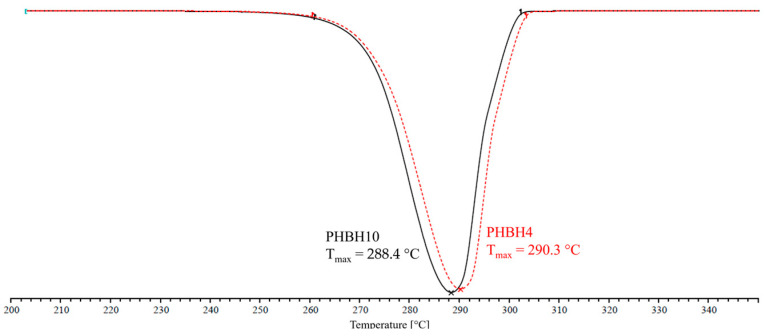
First-order derivative (DTG) of thermal decomposition (TGA) curves of the PHBH10 (solid black line) and PHBH4 (dotted red line).

The DSC heating traces obtained for both PHBH4 and PHBH10 biopolyesters show only one glass transition temperature *T_g_* and a broad melting endotherm *T_m_* with values characteristic for PHAs samples with a relatively wide molar mass distribution. Moreover, the Δ*H_m_* values observed for the tested high molecular mass biopolyester studied are much lower than for poly(hydroxybutyrate) PHB biopolyester of similar molar mass (101.2 J g^−1^) [38]. Samples of the PHBH4T and PHBH10T oligomers were characterized by a lower *T_g_*, *T_m_* and Δ*H_m_* compared to the high molar mass PHBH biopolyesters, which is typical for oligomeric PHAs [39]. Moreover, the determined melting enthalpy values were higher for the PHBH4 and PHBH4T samples compared to the PHBH10 and PHBH10T samples. It can therefore be assumed that the chains of PHBH4 biopolyester and the PHBH4T oligomers obtained from it are characterized by higher ordering, probably due to the lower content of HH units in the biopolyester chains. The decomposition temperatures *T_max_* determined by the TGA method were similar for both samples of high molecular weight biopolyester, PHBH4 and PHBH10, and equaled 290.3 °C and 288.4 °C, respectively (Figure 2).

**Figure 3:**
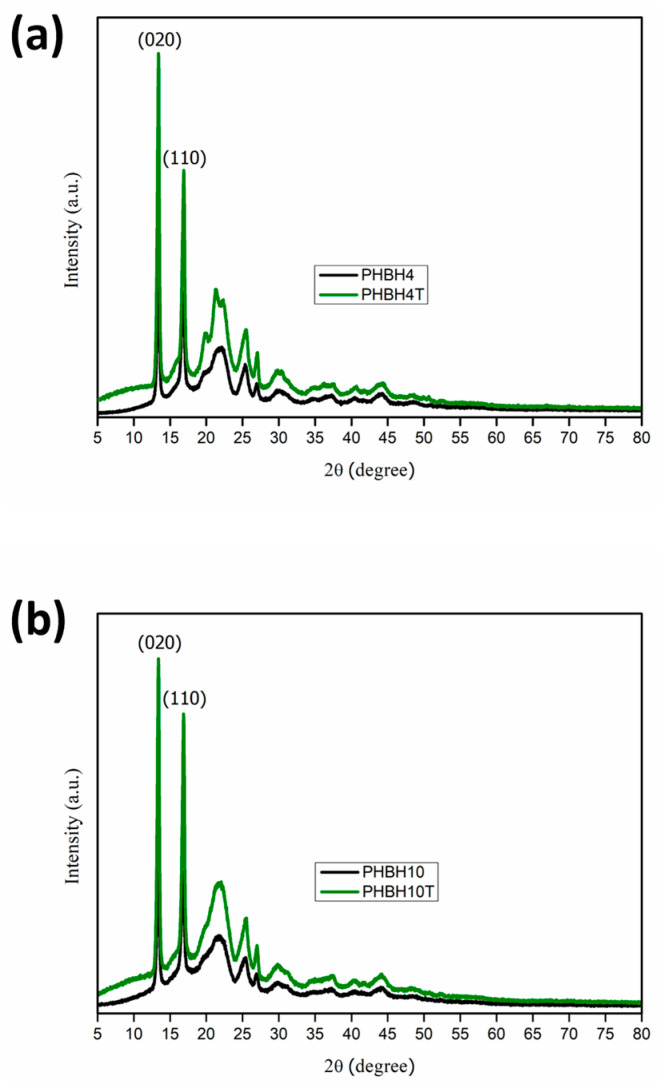
X-ray diffraction patterns of the biopolyesters and respective oligomers obtained via thermal degradation: (**a**) PHBH4 and PHBH4T and (**b**) PHBH10 and PHBH10T.

In the next step, the crystal structure of the studied PHBH biopolyester samples were examined using the X-ray diffraction technique. The X-ray diffraction patterns obtained for the PHBH biopolyester samples before and after thermal degradation are shown in the Figure 3. The presence of sharp peaks with a visible amorphous halo indicates partial crystallinity of the studied PHBH copolyesters. It can be seen that after thermal degradation the intensity of the (020) and (110) peaks increases, indicating the presence of preferred orientation. It should also be noted that the background increases in the samples after thermal degradation, which indicates an increase in the amorphous phase content (Figure 3). This is consistent with the DSC results. In some cases, the crystallites organize in a specific crystallographic direction, which may be due to the specific growth and thermal history of the samples [40,41]. Usually, for the samples with preferred orientation, a significant change in the intensity of the diffraction peaks is observed [42]. However, considering the shape of the X-ray diffraction patterns obtained for thermally degraded samples, to avoid errors, the degree of crystallinity of the tested samples using the peak decomposition method was not determined.

**Figure 4:**
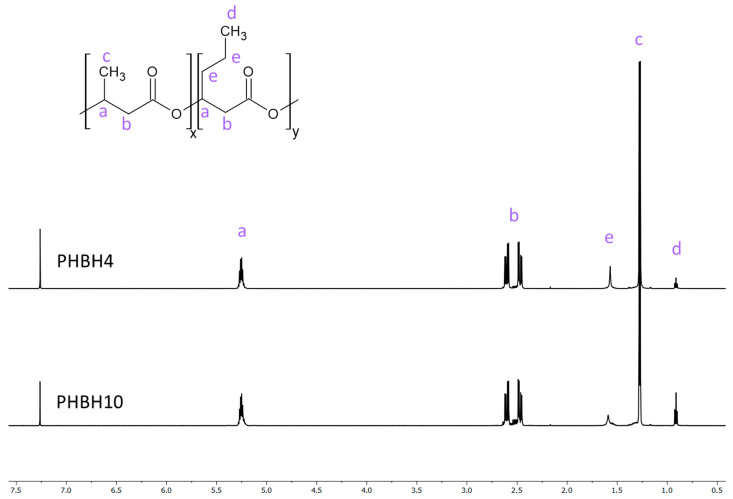
^1^H NMR spectrum of PHBBH4 and PHBH10 biopolyesters.

The specific applications of biopolyesters with defined molecular architecture and properties require detailed and unambiguous information about their structures. Initially, the chemical structures of the PHBH4 and PHBH10 high molar masses biopolyester samples studied were determined by ^1^H NMR analysis and the respective spectra are presented in Figure 4.

The ^1^H NMR spectra of both PHBH4 and PHB10 samples revealed the presence of signals corresponding to the protons of 3-hydroxybutyrate (HB) and 3-hydroxyhexanoate (HH) repeating units [43]. In particular, characteristic signals at δ = 0.91 (d) and 1.59 (e) ppm corresponded to the protons 3-hydroxyhexanoate units (of methyl and methine groups, respectively). The additional signals visible on the spectra located at 1.28 ppm (c); 2.50 ppm (b), and 5.26 ppm (a) constitute a sum of the protons of HB and HH units. The chemical composition of the poly(3-hydroxybutyrate-*co*-3-hydroxyhexanoate) biopolyester samples studied was calculated based on the integral value of the signals corresponding to methyl group protons of HB (c) and HH (d) units, respectively. The copolymer composition of the PHBH4 sample was estimated as 4 mol% of HH units and 96 mol% of HB units, while the composition of PHBH10 was equal 10 mol% of HH units and 90 mol% of HB units.

**Figure 5:**
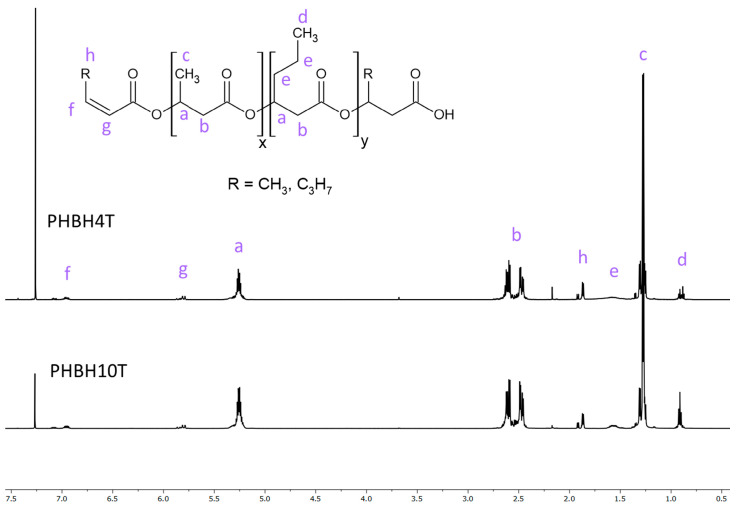
^1^H NMR spectrum of PHBBH4T and PHBH10T oligomers obtained via thermal degradation.

The ^1^H NMR spectra of PHBH4T and PHBH10T low molar mass oligomers (presented in Figure 5) revealed the presence of characteristic signals corresponding to the protons of HB and HH repeating units. Furthermore, additional signals from the olefinic end groups at δ = 1.91; 5.82 and 6.95 ppm were observed.

The content of HH units estimated by ^1^H NMR for the PHBH4T and PHBH10T samples was 4 mol% and 10 mol%. This means that the composition of the obtained PHBH4T and PHBH10T oligomers remained unchanged compared to the tested high molar mass PHBH4 and PHBH10 copolymer samples (see Table 2).

Taking into account the fact that some of the signals observed in the ^1^H NMR spectra of the tested biopolyesters overlap, to verify the chemical structure and determine the distribution of the co-monomer of the tested copolymers, ESI-mass spectrometry was employed. To adjust the molar masses of the tested PHBH biopolyesters to the measurement capabilities of the spectrometer, controlled thermal degradation of the biopolyesters was carried out (see Scheme 1).

**Figure 6:**
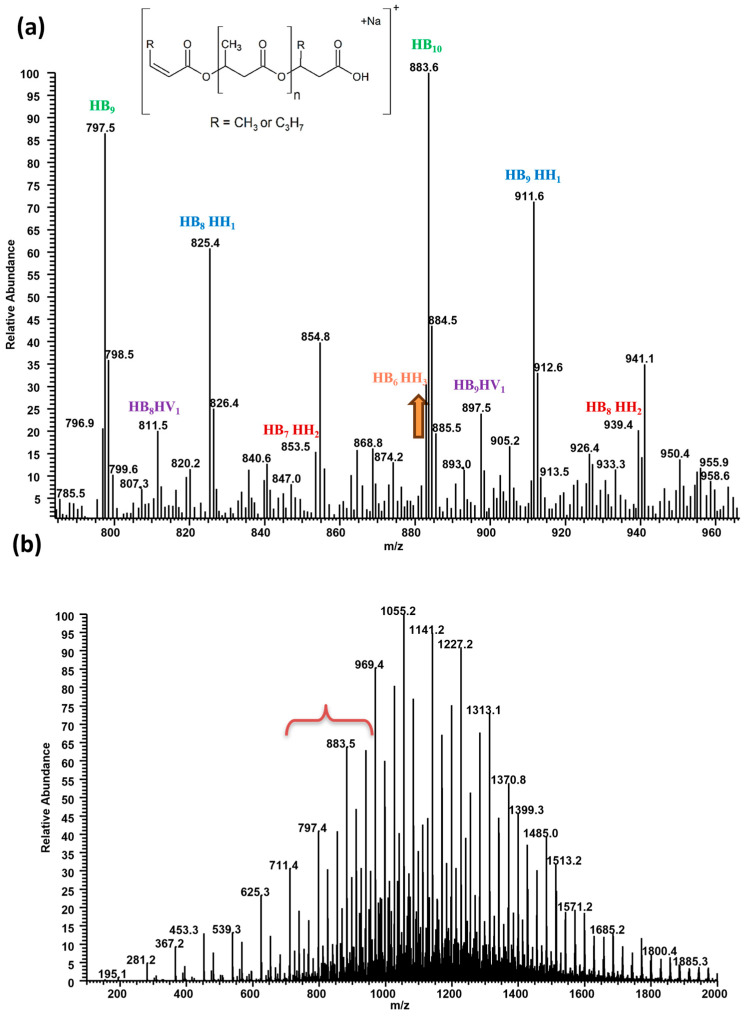
ESI-mass spectra (positive ion mode) of PHBH10T low molecular weight oligomers (**a**) and expanded ESI-MS spectra (positive ion mode) in the mass range 785 < *m/z* < 965 (**b**); The bracketed area represents an enlargement of the ESI-MS spectrum (**a**), providing an extended view of specific spectral details (**b**).

The chemical structure at the molecular level and distribution of comonomer units in the tested PHBH copolymer samples were determined based on the analysis of their oligomers using the ESI-MS/MS technique [44,45]. The ESI-mass spectra of PHBH10T oligomers are presented in Figure 6.

The ESI mass spectrum of PHBH10T oligomers was obtained in positive ion mode. The resulting spectrum displays singly charged ions grouped into clusters, representing biopolyester oligomers. These clusters are separated based on their varying chemical composition and degree of oligomerization, and occur regularly in the mass spectrum as shown in Figure 6. The mass difference between ions within each cluster is 28 Da, corresponding to the difference in molecular masses of individual HH and HB comonomer units in the PHBH biopolyester oligomers.

The observed mass difference between the most intense peaks in neighboring clusters was 86 Da. Based on the mass assignments of the peaks, the spectrum represents sodium adducts of biopolyester oligomers, terminated with carboxyl and unsaturated end groups, as illustrated in Figure 6a. The composition of the ions belonging to the 9-mer and 10-mer clusters is shown in Figure 6a. The expanded region of the mass spectrum in Figure 6a displays two oligomer clusters (9-mer and 10-mer). Each cluster consists of sodium adducts of 3-hydroxybutyrate oligomers and HBHH co-oligoesters containing up to three HH units. These oligoesters are terminated by unsaturated and carboxyl end groups, as illustrated in Figure 6a. Additionally, less intense signals corresponding to oligoesters composed of HB and HV units, also terminated with unsaturated and carboxyl end groups, were identified.

**Figure 7:**
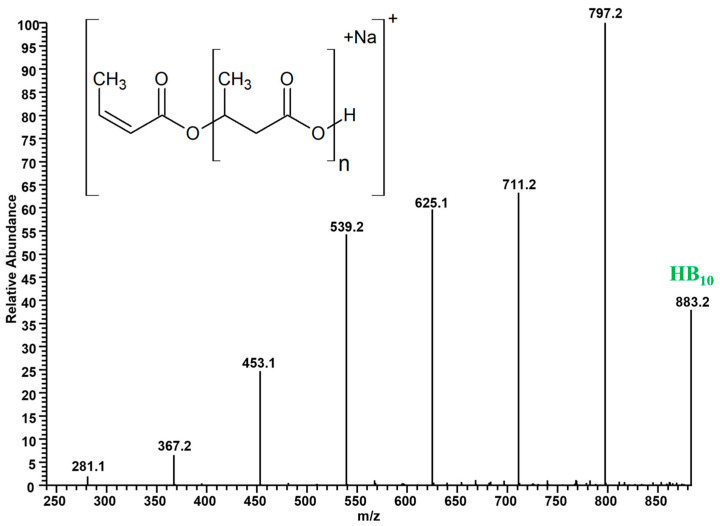
ESI-MS/MS spectrum obtained for sodium adduct of [HB_10_ + Na]^+^ at *m/z* 883.

**Figure 8:**
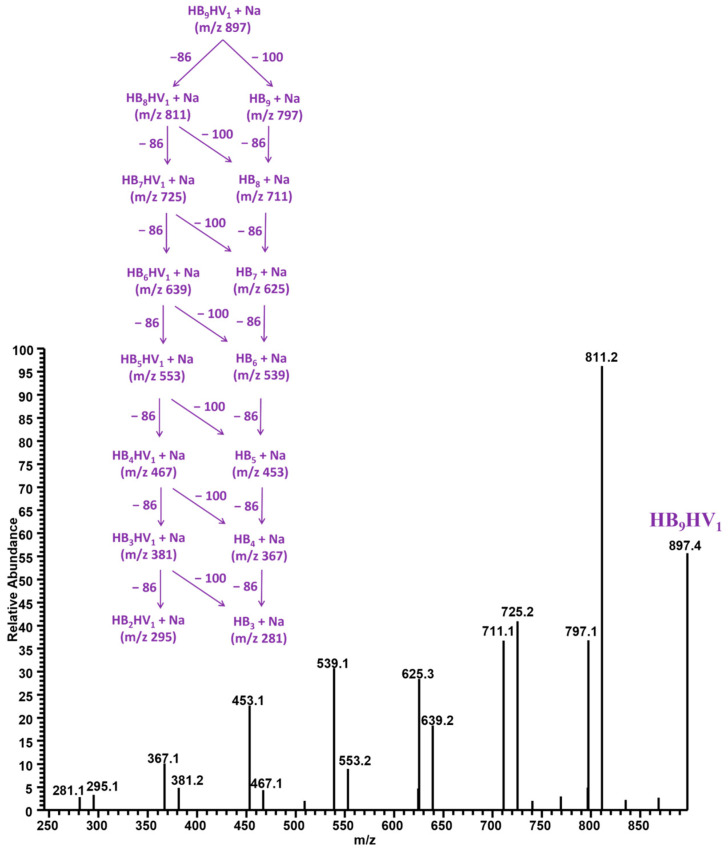
ESI-MS/MS spectrum for the sodium adducts of [HB_9_HV_1_ + Na]^+^ at *m/z* 897.

**Figure 9:**
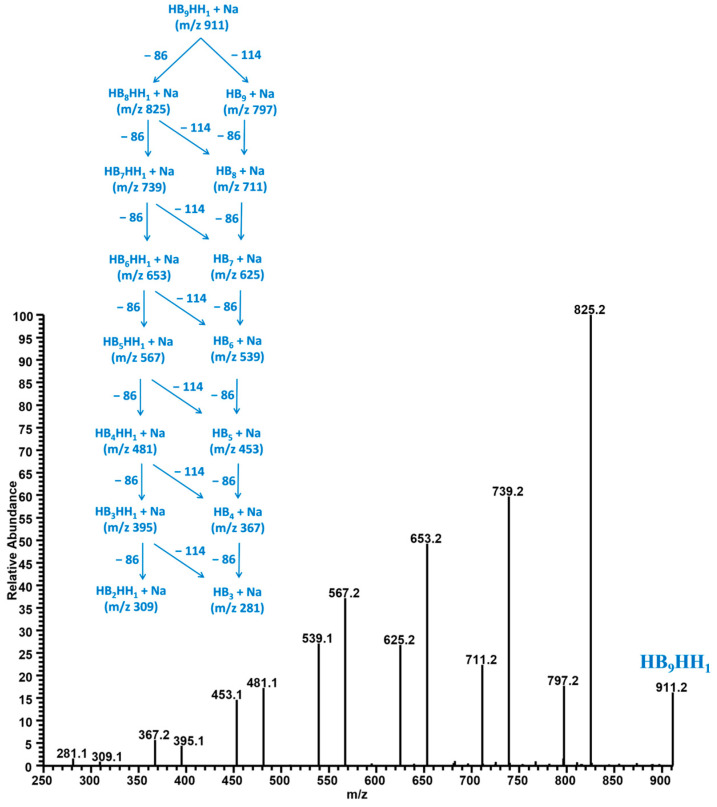
ESI-MS/MS spectrum for the sodium adduct of [HB_9_HH_1_ + Na]^+^ at *m/z* 911.

**Figure 10:**
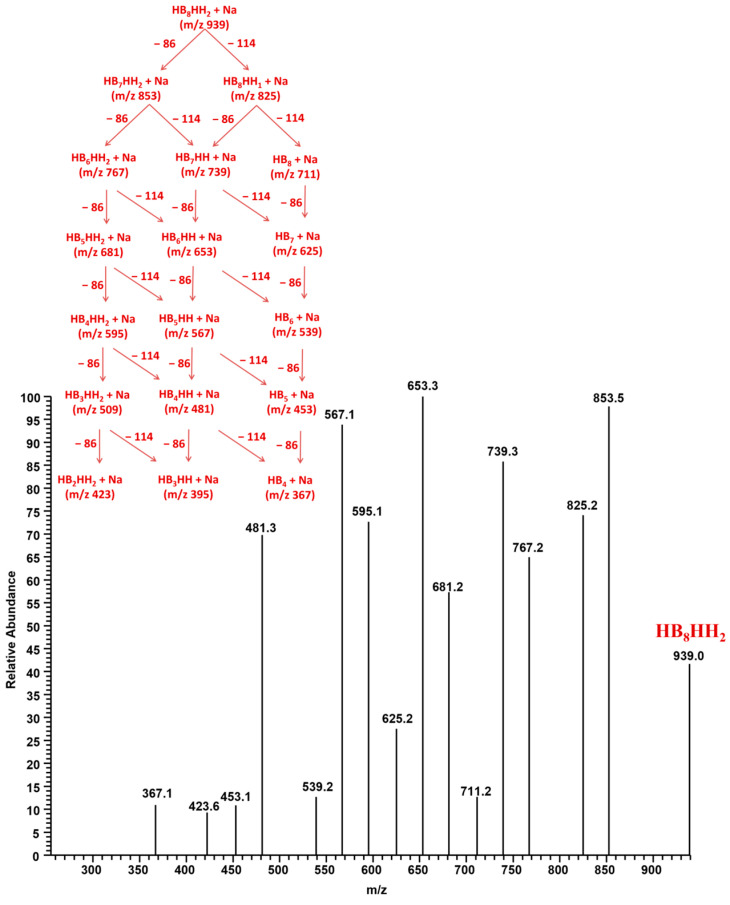
ESI-MS/MS spectrum obtained for sodium adducts of HB_8_HH_2_ at *m/z* 939.

To verify the chemical structure and distribution of comonomer units within the individual oligomer chains of the PHBH10T sample, multistage MS/MS experiments were conducted on selected sodium adduct co-oligoesters at *m/z* 883 [HB_10_ + Na]^+^ (Figure 7), *m/z* 897 [HB_9_HV_1_ + Na]^+^ (Figure 8), *m/z* 911 [HB_9_HH_1_ + Na]^+^ (Figure 9), and *m/z* 939 [HB_8_HH_2_ + Na]^+^ belonging to one of the clusters consisting of 10 comonomer units (Figure 10). Our previous studies on the fragmentation of selected individual PHA oligomer ions in a mass spectrometer have shown that the main mechanism causing the fragmentation of these oligomer ions, through ester bond cleavage, is the β-hydrogen rearrangement [32,44,46].

Figure 7 shows the ESI-MS/MS spectrum of the sodium adduct of oligo(3-hydroxybutyrate) with crotonate and carboxyl end groups. In this spectrum, the breaking of ester bonds along the oligomer chain produces a one series of oligo(3-hydroxybutyrate) fragment, all featuring carboxyl and crotonate end groups.

The ESI-MS/MS spectrum of the sodium adduct of oligo[(3-hydroxybutyrate-*co*-3-hydroxyvalerate] at *m/z* 897 (HB_9_HV_1_) was generated by the loss of either crotonic acid (86 Da) or 2-pentenoic acid (100 Da) in the first step (Figure 8). Subsequent cleavage of ester bonds along the oligoester chains produced two sets of product ions, terminated by carboxyl and crotonate or carboxyl and 2-pentenoate end groups.

Similarly, the ESI-MS/MS spectrum of the sodium adduct of the HB_9_HH_1_ co-oligoester at *m/z* 911 was obtained through random cleavage of ester bonds along the co-oligoester chains (Figure 9). This resulted in two sets of products oligomers, each terminated by either carboxyl and crotonate end groups or carboxyl and 2-hexenoate end groups.

The results presented in Figure 10 of the MS^2^ fragmentation experiment for the sodium adduct of the HB_8_HH_2_ co-oligoester at *m/z* 939 indicate that this sodium adduct corresponds to a mixture of macromolecules containing two randomly positioned HH units along the oligomers chains. Initially, two fragment ions are formed: HB_7_HH_2_ and HB_8_HH_1_ (see the accompanying scheme in Figure 10). Subsequently, a set of clusters is produced, containing three fragment ions of the same degree of oligomerization but differing in their content of HH units due to the successive loss of crotonic acid or 2-hexenoic acid (114).

The results of the fragmentation experiments confirmed that the isolated ions represent sodium adducts of single oligoester chains composed mainly of HB and HH units terminated with carboxyl and unsaturated end groups. A certain amount of oligoester chains composed of HB and HV co-monomeric units was also detected. Moreover, the paths of the formed fragmentation products observed in the ESI-MS/MS spectra indicate that the HB, HH, and HV co-monomeric units are randomly distributed along the PHBH biopolyester chains.

The results of the research conducted in this study deepen our understanding of the relationship between molecular architecture and the thermal behavior of PHA biopolyesters. This knowledge can support the design and development of more efficient and environmentally friendly biopolyesters for new applications. The non-toxic nature, exceptional biocompatibility, and ability of microbial biopolyesters to degrade in vivo make this group of biopolymers promising materials for various biomedical applications. For instance, PHBH biopolyesters are gaining increasing attention for their potential as scaffolds in tissue engineering. However, several limitations should be acknowledged. For example, in specific medical applications, biopolyesters often do not have sufficient thermomechanical properties, and do not show adequate degradation kinetics and cytocompatibility. To enhance their potential in medicine, PHAs are subjected to physical or chemical modifications. Our study did not include degradation kinetics as well as biological tests of the studied PHBH. This may be a limitation, because understanding degradation processes and biocompatibility with respect to the corresponding substances is crucial for real-world applications of PHBH biopolyesters, especially in fields such as medicine. We hope that our planned future studies on these factors can help build a more comprehensive understanding of the durability and performance of biomaterials developed using PHBH biopolyesters for specific medical applications.

## 4. Conclusions

In conclusion, this study provides valuable insights into the molecular structure and thermal degradation behavior of two high-molecular-weight biopolyesters, poly(3-hydroxybutyrate-*co*-3-hydroxyhexanoate) (PHBH), produced under the Bluepha^®^ trademark. Proton NMR analysis, used to evaluate the structure of the PHBH samples, confirmed the proton signals characteristic of repeating 3-hydroxybutyrate (3-HB) and 3-hydroxyvalerate (3-HV) units. Based on the ^1^H NMR spectra, the chemical composition of the PHBH4 sample was estimated to be 4 mol% HH units and 96 mol% HB units, while the composition of PHBH10 was 10 mol% HH and 90 mol% HB.

Additionally, to further verify the structure of the PHBH biopolyester samples, mass spectrometry was applied, as this method is known to provide more detailed information on the structure and sequential distribution of comonomer units along the copolymer chains than ^1^H NMR alone. The chemical structure at the molecular level and the distribution of comonomer units in the analyzed PHBH copolymer samples were determined based on oligomer analysis using the ESI-MS/MS technique. These studies also revealed that, in addition to polymer chains composed of HB and HH, the PHBH biopolyester samples contained some polyester macromolecules composed of HB and HV units. The fragmentation pattern of the selected molecular ions confirmed that the HB, HH, and HV comonomer units are randomly distributed along the PHBH biopolyester chains.

The significance of the studied PHBH biopolyesters lies in the fact that their thermal properties, such as *T_g_*, *T_m_*, and melting enthalpy Δ*H_m_*, are more favorable than those of poly(hydroxybutyrate) (PHB), indicating broader applications for these biopolyesters compared to the PHB homopolymer. Moreover, the presence of HH units significantly affects both the structural and thermal properties of PHBH biopolyesters, with higher HH content leading to reduced chain ordering, as demonstrated by DSC and XRD analyses.

## Figures and Tables

**Table 1:** Number average molar mass (*M_n_*), weight average molar mass (*M_w_*), dispersity (*M_w_/M_n_*), composition of the PHBH4 and PHBH10 copolymers and respective oligomers obtained via thermal degradation.

Sample Name	*M_n_* *	*M_w_* *	*M_w_*/*M_n_* *	Composition ** mol%HB/mol%HH
PHBH4	412,000	863,000	2.09	96/4
PHBH10	305,000	665,00	2.18	90/10
PHBH4T	1100	2200	2.00	96/4
PHBH10T	1300	2900	2.22	90/10

* Estimated by GPC; ** chemical composition of PHBH in mol% estimated from ^1^H NMR, HB (hydroxybutyrate) and HH (hydroxyhexanoate).

**Table 2:** DSC results of the PHBH4 and PHBH10 biopolyesters and resulting PHBH4T and PHBH10T oligomers obtained via thermal degradation.

Sample Name	*T_g_* (II Run) [°C]	*T_m_* (I Run) [°C]	Δ*H_m_* (J g^−1^)
PHBH4	3.7	122.4/149.7	68.2
PHBH10	1.3	114.0	48.8
PHBH4T	−10.0	89.1/108.2/125.5	57.7
PHBH10T	−7.4	94.8/113.7/122.2	46.1

*T_g_*—glass transition temperature, *T_m_*—melting temperature, Δ*H_m_*—melting enthalpy.

## Data Availability

The original contributions presented in the study are included in the article, further inquiries can be directed to the corresponding author.
